# Wearable Movement Sensors for Rehabilitation: From Technology to Clinical Practice

**DOI:** 10.3390/s21144744

**Published:** 2021-07-12

**Authors:** Gerrit Ruben Hendrik Regterschot, Gerard M. Ribbers, Johannes B. J. Bussmann

**Affiliations:** 1 Department of Rehabilitation Medicine, Erasmus University Medical Center Rotterdam, P.O. Box 2040, 3000 CA Rotterdam, The Netherlands; g.ribbers@erasmusmc.nl (G.M.R.); j.b.j.bussmann@erasmusmc.nl (J.B.J.B.); 2 Rijndam Rehabilitation, Westersingel 300, 3015 LJ Rotterdam, The Netherlands

Motor disorders are a common and age-related problem in the general community. Therefore, medical rehabilitation often focuses on reducing the burden of motor disorders. With an aging society, the burden of motor disorders is expected to grow, while the healthcare capacity is not expected to match this growth. For this reason, there is an urgent need to optimize medical rehabilitation of motor disorders both in terms of effectiveness and efficiency. 

The rapid innovations in wearable movement sensors in recent years may provide an opportunity to translate these innovations into the field of motor rehabilitation. Wearable movement sensors can provide objective and precise measurements of the quantity and quality of physical activities, body postures, and movements in clinical as well as normal daily life environments, thereby providing clinicians with data that can be used to guide, personalize, and optimize therapy. Since wearable sensors are portable, inexpensive, unobtrusive, and also have the ability to provide information that is unique and cannot be obtained otherwise (e.g., by standardized clinical tests or questionnaires), they have an enormous potential for the tracking of patient functioning and recovery during motor rehabilitation. In addition, wearables can play a crucial role in the existing tendency towards at-home monitoring and treatment, and in substituting more complex measurement devices, such as camera systems.

Despite their potential to optimize motor rehabilitation, wearable movement sensors are relatively scarcely applied in rehabilitation of motor disorders. Important challenges remain, such as the development of reliable and valid wearable movement sensors in clinical populations and free-living environments, barriers in the deployment of wearable movement sensors in clinical care, development and optimization of innovative sensor configurations and data analysis techniques (such as machine learning-based algorithms that enable detection of specific activities and movements in free-living conditions), and the development of disease-specific sensor-based outcome measures that are relevant and interpretable by patients and clinicians.

This Special Issue, entitled “Wearable Movement Sensors for Rehabilitation: From Technology to Clinical Practice”, aims to facilitate the application of wearable movement sensors in clinical practice. It intends to explore the opportunities for the application of wearable movement sensors in motor rehabilitation.

A total of 17 papers are published in this Special Issue. These papers mainly focus on the following topics: algorithm development, technical validation, clinical validation, monitoring of physical behavior in daily life conditions, and implementation in motor rehabilitation. Hereafter, we provide a brief overview of each paper. 

Yang et al. (2020) [1] developed an online gait-planning algorithm based on sensing signals to enable balance control during exo-skeleton assisted walking with crutches in spinal cord patients. Results from this pilot study in healthy adults indicate that the developed online gait-planning algorithm can plan the landing point of the swing leg to improve balance control during exo-skeleton assisted walking. The algorithm may be useful to improve balance control during exoskeleton assisted walking in spinal cord injury patients and reduce the need of using crutches.

Sy et al. (2020) [2] present a novel Lie group constrained extended Kalman filter to estimate lower limb kinematics with a minimal sensing solution during different physical activities (e.g., walking). Healthy adults performed an activity protocol with three inertial measurement unit (IMU) sensors, placed on the pelvis and on both ankles. Results showed relatively small errors for the knee and hip joint angles compared to an optimal motion capture system, indicating the validity of the algorithm. This paper contributes to the development of a sensor-based method that enables comfortable and long-term monitoring of lower limb kinematics in rehabilitation patient populations.

Fadel et al. (2020) [3] propose a new algorithm that quantifies the characteristics of walking based on a hip-worn accelerometer in a more detailed manner than activity counts. The algorithm uses the fast Fourier Transform to obtain periodic characteristics of walking, and it reduces the dimensionality of the raw sensor data into a form that retains details of the original signal while enabling existing statistical methods for analyses. The paper serves as a proof of concept for how researchers can extract the walking characteristics from sensor data and investigate the association with relevant health-related outcomes in rehabilitation. An example is provided of a study that investigates the associations of walking spectra obtained from the fast-paced 400 m walk with age and BMI in older adults.

Roossien et al. (2021) [4] developed a sensor-based method for the measurement of lumbar load. The method consists of six IMU sensors on the upper and lower arms, sternum, and pelvis. Lumbar load is quantified as the net moment around the L5/S1 intervertebral body, estimated using a method that is based on artificial neural networks. The validity of the sensor-based method was supported in healthy adults since the differences in the estimated lumbar load were consistent with the perceived intensity levels and the character of the work tasks. The method may be used to monitor lumbar load in people with musculoskeletal disorders such as lower back pain, to assess muscular overload during rehabilitation, and to help clinicians to tailor treatments.

Bravi et al. (2021) [5] investigated the validity of a sensor-based system for shoulder range of motion assessment in cervical spinal cord injury patients. The sensor-system consists of two IMU sensors placed on the wrist and upper arm. Patients and healthy controls performed four shoulder movements. Every movement was evaluated with a goniometer and with the IMU system at the same time. The validity of IMU system was partially confirmed, since relative agreement between the IMU system and goniometer was high but absolute agreement was relatively low. The proposed IMU system may be a potential tool for monitoring shoulder range of motion in patients with cervical spinal cord injury.

Tak et al. (2020) [6] presented a new sensor-based method for the measurement of knee, hip and spine joint angles during the single leg squat. The sensor system consists of four IMU sensors placed on the trunk, pelvis, upper leg, and lower leg. In this study, healthy adults performed single leg squats. Results showed high correlations between the joint angles measured with the sensor system and an optical reference system, indicating the validity of the sensor system. The sensor method is potentially relevant for monitoring and optimizing lower extremity kinematics during rehabilitation interventions.

Ahmadi et al. (2020) [7] investigated the accuracy of group, group-personalized, and fully-personalized machine learning physical activity classification models in children with cerebral palsy. Models were trained and tested using accelerometer data from the hip, wrist, and ankle. To assess the validity, the classification accuracy was evaluated and compared in a laboratory trial and a simulated free-living trial with 38 children while wearing a wrist-worn accelerometer. Results showed that group-personalized and fully-personalized Random Forest activity classification models provide a more accurate recognition of physical activity in children with CP than “one-size-fits-all” group models.

Labarierre et al. (2020) [8] performed a systematic review to identify and summarize studies in which motion sensors and machine learning algorithms have been used to adapt the behavior of orthotic/prosthetic devices to user locomotion mode (e.g., stair ascent/descent, walking on flat floor). Results showed that classification accuracies were, in general, very high in healthy people and people with unilateral transtibial and transfemoral amputation. These findings support the validity of sensor methods and machine learning algorithms to recognize locomotion mode. 

Yang et al. (2020) [9] developed a sensor-based method to estimate relative 3D orientations between finger tips and the dorsal side of the hand with inertial motion sensors but without magnetometers to avoid magnetic disturbance. The method consists of one sensor on the dorsal side of the hand, and one on the most distal finger segment. Results in three healthy adults show that errors in relative orientation between fingers and hand are relatively small during hand movements and during a functional water-drinking task. The sensor method is potentially useful for clinical assessments during stroke rehabilitation.

Prasanth et al. (2021) [10] performed a systematic review of sensor-based methods applied for real-time gait analysis. Inertial measurement units on the shank and foot are most often used for gait analysis in combination with threshold or peak identification methods for gait detection. Less than one third of the sensor-based methods for gait analysis were validated on pathological gait data. For clinical gait assessments, a combination of inertial measurement units and rule-based methods are recommended as an optimal solution.

Regterschot et al. (2021) [11] investigated to what extent arm use measurements with wrist-worn accelerometers in stroke patients are affected by whole-body movements, such as walking. Wrist-worn accelerometers are often applied to measure arm use after stroke. They measure arm use by recording all arm movements, including non-functional arm movements due to whole-body movements. Results of the study show that whole-body movements substantially increase cross-sectional arm use outcomes when not correcting arm use data for whole-body movements, thereby threatening the validity of arm use outcomes and measured arm use changes.

Zhou et al. (2020) [12] evaluated a sensor-based method to classify fallers from non-fallers based on spatial-temporal gait characteristics. Wearable sensors were placed on both ankles and the lower back. A partial least square discriminant analysis was used to classify fallers and non-fallers based on gait features derived from the sensor data. Results showed that fallers differed from non-fallers in gait patterns. The presented sensor-based method may be useful in rehabilitation to identify persons with a high fall risk and to monitor the effects of interventions on fall risk. 

Mazzarella et al. (2020) [13] investigated in this pilot study whether a 3D motion capture system can detect changes over time in pre-reaching and reaching behaviors in infants with perinatal stroke and cerebral palsy. Results showed that spatiotemporal characteristics of upper extremity movements measured with a 3D motion capture system change over time in infants with typical development, cerebral palsy and perinatal stroke, with potential differences between infants with typical development and cerebral palsy. This study shows the potential of wearable sensors for measuring characteristics of upper extremity movements in infants with perinatal stroke and cerebral palsy.

Fleiner et al. (2021) [14] investigated the association between physical behavior and subjectively-rated circadian chronotypes in older adults. Physical activity was measured in 81 older adults during one week with a motion sensor on the lower back and the wrist. Results showed that the timing of mobility-related activity is associated with subjectively-rated chronotypes in older adults. The presented sensor-based method may provide a useful approach for early detecting and tailoring the treatment of circadian disruptions in rehabilitation populations.

Hofstad et al. (2020) [15] measured the number of consecutive steps and walking bouts in persons with a lower limb amputation using three accelerometers: one in each trouser pocket and one on the sternum. Measurements were performed for two consecutive days in 20 persons with a lower limb amputation and 10 age-matched controls. Results showed that objectively measured mobility was highly affected in persons with an amputation and that self-reported mobility did not match with the objective sensor-based measurements. This study recommends the use of accelerometers to measure mobility in persons with a lower limb amputation.

Lang et al. (2020) [16] discussed the major barriers for the application of wearable movement sensors in motor rehabilitation and proposed benchmarks for the implementation of sensors in clinical practice. Barriers in the clinic are the busy clinical environment and the lack of realization of the value of the information that can be obtained with sensors. Technology-related barriers include: (1) sensor systems that are inaccurate for many patient populations; (2) sensor systems that are not user-friendly for clinicians and/or patients; (3) the lack of published data regarding reliability and clinical validity of sensor systems.

Braakhuis et al. (2021) [17] explored the use, perspectives, and barriers to wearable activity monitoring in day-to-day stroke care routines amongst physical therapists. Results of the online survey showed that 27% of the respondents were using activity monitoring, and the concept of remote activity monitoring was perceived as useful. The identified barriers to clinical implementation were lack of skills and knowledge of patients, financial constraints, and not knowing what type of monitor to apply.

This Special Issue shows a range of potential opportunities for the application of wearable movement sensors in motor rehabilitation. However, the papers surely do not cover the whole field of physical behavior monitoring in motor rehabilitation. Most studies in this Special Issue focused on the technical validation of wearable sensors and the development of algorithms. Clinical validation studies, studies applying wearable sensors for the monitoring of physical behavior in daily life conditions, and papers about the implementation of wearable sensors in motor rehabilitation are under-represented in this Special Issue. Studies investigating the usability and feasibility of wearable movement sensors in clinical populations were lacking. We encourage researchers to investigate the usability, acceptance, feasibility, reliability, and clinical validity of wearable sensors in clinical populations to facilitate the application of wearable movement sensors in motor rehabilitation.
